# Quality indicators for lifestyle or behavioural management for the primary prevention of cardiovascular disease in primary care: a systematic review

**DOI:** 10.3399/BJGPO.2025.0018

**Published:** 2025-12-19

**Authors:** Kiran Bam, Beilei Lin, Muideen T Olaiya, Dominique A Cadilhac, Julie Redfern, Mark R Nelson, Lauren M Sanders, Nadine E Andrew, Vijaya Sundararajan, Lisa Murphy, Monique F Kilkenny

**Affiliations:** 1 Stroke and Ageing Research, Department of Medicine, School of Clinical Sciences at Monash Health, Monash University, Clayton, Australia; 2 The Nursing and Health School, Zhengzhou University, Henan, China; 3 Stroke and Critical Care Research Theme, The Florey, University of Melbourne, Heidelberg, Australia; 4 School of Health Sciences, Faculty of Medicine and Health, University of Sydney, Sydney, Australia; 5 The George Institute for Global Health, University of New South Wales, Sydney, Australia; 6 Institute for Evidence-Based Healthcare, Bond University, Robina, Australia; 7 Menzies Institute for Medical Research, University of Tasmania, Hobart, Australia; 8 School of Public Health and Preventive Medicine, Monash University, Melbourne, Australia; 9 Department of Neurosciences, St Vincent’s Hospital Melbourne, Fitzroy, Australia; 10 Department of Medicine, Melbourne Medical School, University of Melbourne, Parkville, Australia; 11 Peninsula Clinical School, School of Translational Medicine, Monash University, Frankston, Australia; 12 National Centre for Healthy Ageing, Monash University, Frankston, Australia; 13 Department of Medicine, University of Melbourne, Parkville, Australia; 14 Stroke Foundation, Melbourne, Australia

**Keywords:** quality indicators, cardiovascular risk factors, heart disease risk factors, cardiovascular diseases, primary prevention, primary health care

## Abstract

**Background:**

Monitoring lifestyle or behavioural risk factors using quality indicators is critical for the primary prevention of cardiovascular disease (CVD).

**Aim:**

To summarise indicators for monitoring lifestyle risk factors for the primary prevention of CVD.

**Design & setting:**

A systematic review of quality indicators in primary care.

**Method:**

Four research databases (Ovid MEDLINE, Ovid Embase, CINAHL Plus, and Scopus) and grey literature were searched to identify articles (indicator sets) used to monitor lifestyle risk factors. Articles were assessed for methodological quality using the Appraisal of Indicators through Research and Evaluation (AIRE) instrument. Articles with strong methodological quality, scoring ≥50% in each domain (that is, relevance, stakeholder involvement, scientific evidence, and usage) were included. Indicators were categorised into assessment of lifestyle risk factors or advice on healthy lifestyle.

**Results:**

We identified 39/282 (14%) articles including indicators to monitor lifestyle risk factors from a full-text review. Of these, 19 (49%) articles with strong methodological quality, comprising 90 unique indicators, were included. Most of the indicators were on assessing smoking status (21%), body weight (18%), advice on smoking cessation (13%), immunisation (9%), and advice on physical activity (8%). Assessment of alcohol consumption (3%) and healthy eating (2%) were the least reported. When comparing assessment versus advice indicators, we found gaps in monitoring smoking status (41% assessment versus 27% advice) and body weight (35% versus 14%). Notably, there were more indicators for advice on (16%) than assessment of (4%) healthy eating.

**Conclusion:**

We identified several indicators for the monitoring of lifestyle risk factors. However, there is a need to ensure an appropriate mix of indicators on assessment versus advice.

## How this fits in

Monitoring lifestyle or behavioural risk factors using quality indicators is critical for the primary prevention of cardiovascular disease. This study identified a large number of quality indicators (*n* = 90 extracted from 19 articles) for the assessment of lifestyle risk factors and advice on healthy lifestyle. However, there is a need for an appropriate balance of assessment of lifestyle risk factors, followed by healthy advice-related quality indicators. These indicators need to be harmonised to ensure methodological and country contextual fitness.

## Introduction

Cardiovascular diseases (CVDs), including heart disease and stroke, are a leading cause of death and disability worldwide, resulting in significant costs to the health system and overall economy.^1^ With the current burden of CVD, global medical costs for CVD management are projected to exceed $1 trillion per year by 2030.^2^ Therefore, primary prevention of CVD is a critical public health priority.

Lifestyle management is an effective strategy and is considered a first-line recommendation in clinical practice guidelines for the primary prevention of CVD.^3–5^ Lifestyle management involves monitoring of lifestyle risk factors (for example, assessment of smoking status) and adherence to a healthy lifestyle (for example, smoking cessation) in primary care.^5,6^ Adopting a healthy lifestyle reduces the need for use of prevention medications.^5,7^ Primary care providers are typically encouraged to document process of care including a patient’s clinical profile (for example, blood pressure and smoking status) or prescription of medication to demonstrate that they adhere to clinical practice guidelines.^7,8^

Quality indicators (described as indicators hereafter) are tools used to assess the effectiveness of primary care.^9^ In the context of primary prevention, these indicators (for example, number of patients with smoking status recorded) typically include a numerator (for example, assessment of current smoking status) and denominator (for example, number of patients) to define a performance standard based on local clinical practice guidelines.^10,11^ These indicators should be relevant, validated, and feasible for timely monitoring of lifestyle risk factors.^10,11^ Therefore, there is a need to understand the current monitoring status of processes of care for lifestyle risk factors using indicators for the primary prevention of CVD.^12^ In this review, we aimed to identify and summarise quality indicators being used for monitoring lifestyle risk factors for the primary prevention of CVD.

## Method

### Study design, search strategy, and selection criteria

A systematic review was undertaken to identify articles in which lifestyle management indicators for the primary prevention of CVD were reported. This review is part of a larger systematic review registered in the International Prospective Register of Systematic Reviews (PROSPERO ID number: CRD42022359131). The review followed the Preferred Reporting Items for Systematic Reviews and Meta-Analyses (PRISMA) 2020 guidelines.^13^ Electronic databases were searched to identify journal articles, including Ovid MEDLINE, Ovid Embase, CINAHL Plus, and Scopus. Google and Google Scholar were used to search for grey literature. The search strategy was limited to CVD or stroke or transient ischaemic attack, risk factors, primary prevention, and primary care settings (clinical practice, family practice, or general practice). Search terms included 'cardiovascular disease', 'stroke', 'quality indicators', 'primary prevention', 'primary care', or 'risk factors' (see Supplementary Table S1). These search terms were mapped into the Medical Subject Headings (MeSH), and wildcards (for example, # and $) or truncations (for example, *) were used as appropriate. Articles or grey literature published from January 2010–August 2022 were included in this review. Additional searches and grey literature from countries with similar primary care systems, such as the UK, Australia, and Canada, were updated in October 2023.

### Inclusion and exclusion criteria

We included quality indicator sets reported in articles published in the English language. We focused on indicators related to assessment of lifestyle risk factors or advice on healthy lifestyle in primary care settings from all regions of the world. Articles that did not focus on the primary prevention of CVD or primary care were excluded. Editorials, research letters, case reports, opinion pieces, comments, viewpoints, correspondences, and consensus documents were excluded.

### Article screening

Unique articles (excluding duplicates from EndNote [version 19]) were imported into Covidence for title and abstract screening.^14^ KB, MFK, and MTO screened the titles and abstracts of articles for eligibility, followed by full-text screening, referring to inclusion and exclusion criteria for selecting articles. DAC resolved conflicts between any two authors.

### Data extraction

We extracted data on country, conditions, domains (assessment, advice, or education),^15^ numerator, denominator, and the frequency of monitoring for each quality indicator set (see Supplementary Table S2). Data extraction was undertaken by KB and reviewed by MTO and BL, and MFK or DAC resolved any disagreements.

### Methodological assessment of quality indicator sets

**Figure 1.**
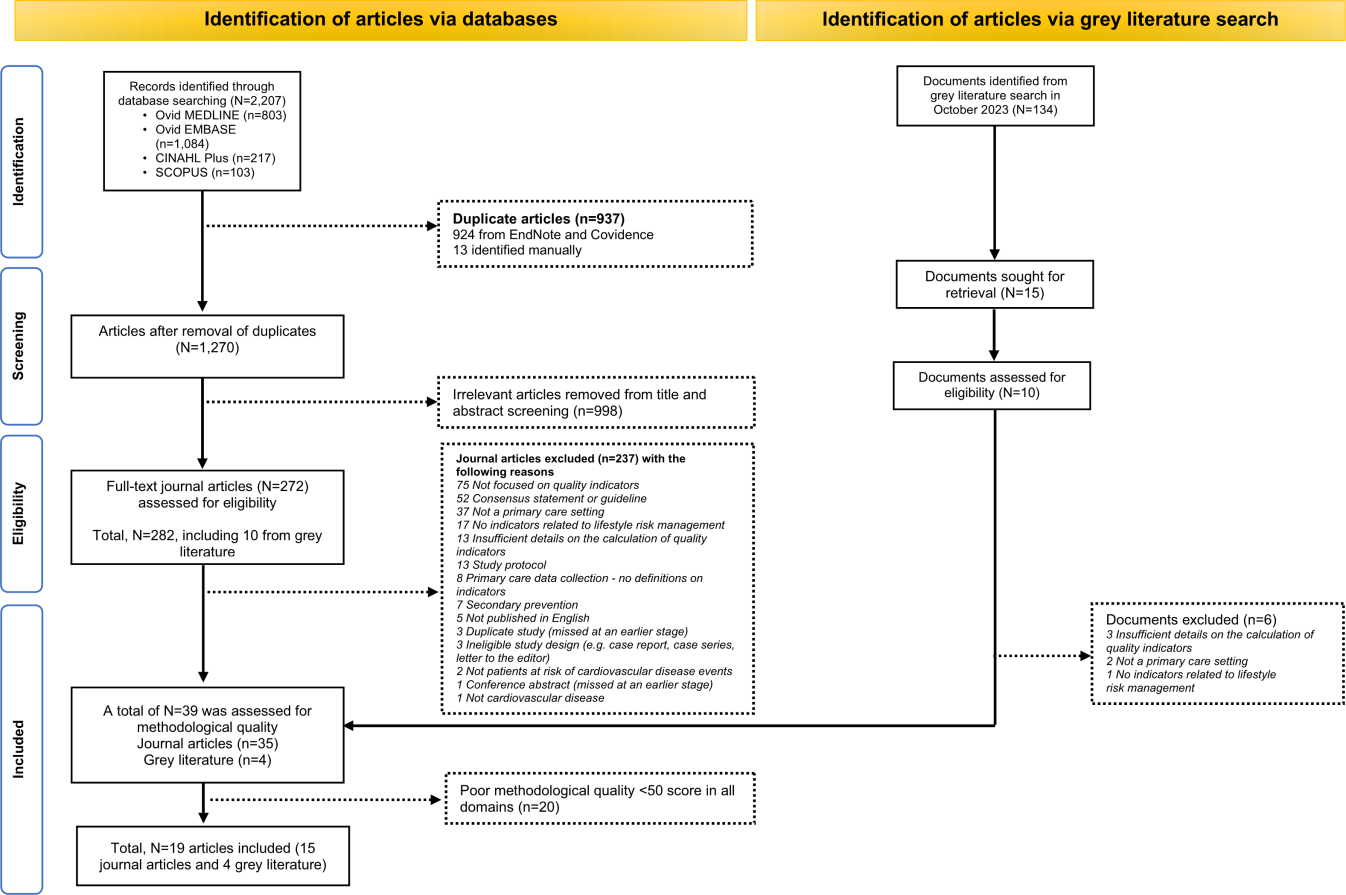
Preferred Reporting Items for Systematic Reviews and Meta-Analyses (PRISMA) 2020 flow diagram including searches of databases and grey literature

Eligible articles were further assessed for methodological quality using the Appraisal of Indicators through Research and Evaluation (AIRE) tool. The AIRE tool comprises the following four domains: a) purpose, relevance, and organisational context; b) stakeholder involvement; c) scientific evidence; and d) additional evidence, formulation, and usage. Each item in the domains was scored on a four-point Likert scale. Standardised scores were calculated, ranging from 0%–100%, with ≥50% representing a strong methodological quality indicator set. This criterion was based on earlier reviews reporting on the appraisal of quality indicators.^16,17^ Articles including indicators set with weak methodological quality were excluded (Figure 1 and Supplementary Figure S1).

### Data analysis and visualisation

Descriptive statistics (frequencies and percentages) were used to summarise the indicators. Pivot tables and heat maps in Microsoft Excel were used for data analysis and visualisation.

## Results

Our search of research databases (*n* = 2207) and grey literature (*n* = 134) yielded 2341 articles. Of these, 282 (12.0%) full-text articles including indicators on the monitoring of lifestyle risk factors were reviewed. Indicators on lifestyle management were reported in 39 (13.8%) articles. Of which, 19 (48.7%) articles with strong methodological quality were included in this review (Figure 1, Table 1).^15,18–35^ Overall, 90 quality indicators were extracted from these 19 articles. These indicators were unique to the country, populations at risk of CVD (categorised based on risk scoring or assessment of multiple risk factors), or disease conditions (for example, hypertension and diabetes). Forty-six indicators (51.1%) were on assessment of lifestyle risk factors,^15,18–20,22–30,33–35^ while 44 (48.9%) were on advice on healthy lifestyle.^15,18,19,21–28,30–32,34,35^

**Table 1.** Characteristics of the included articles (*n* = 19) related to lifestyle management quality indicators (*n* = 90)

Author, year	Country	Conditions^a^	Indicators, *n*	Study design or methodology	Domains	
					Assessment	Advice or education
Aktaa *et al*, 2022^33^	European countries^b^	High risk of CVD	1	Systematic review and Delphi method	√	
Collins *et al*, 2021^20^	Tajikistan	Hypertension	2	Mixed methods	√	
Department of Health, 2019^23^	Australia	No existing risk factor, diabetes	6	Grey literature	√	√
Dixon *et al*, 2015^28^	UK	No existing risk factor, high risk of CVD, diabetes	4	Grey literature	√	√
Honeyford *et al*, 2013^34^	UK	Hypertension, diabetes, COPD, asthma	2	Cross-sectional study	√	√
Huber *et al*, 2020^21^	Switzerland	Risk of CVD	1	Cohort study		√
Khanji *et al*, 2019^19^	Canada	Moderate or high risk of CVD	20	Randomised controlled trial (secondary analysis)	√	√
Kontopantelis *et al*, 2014^24^	UK	Diabetes, hypertension	3	Cohort study	√	√
Lindner *et al*, 2019^30^	US	No existing risk factor	2	Cross-sectional study	√	√
Ludt *et al*, 2013^22^	European countries^c^	High risk of CVD	6	Cross-sectional study	√	√
Ludt *et al*, 2014^35^	European countries^d^	High risk of CVD	8	Cross-sectional study	√	√
NICE, 2023^25^	UK	Hypertension, moderate or high risk of CVD, patients with multiple conditions	5	Grey literature	√	√
Petek *et al*, 2012^15^	Slovenia	Moderate or high risk of CVD, diabetes, no existing risk factor, obesity, smoking, hypertension	18	Literature review and modified Delphi process	√	√
RACGP, 2015^29^	Australia	No existing risk factor	2	Grey literature	√	√
Ralph *et al*, 2013^26^	Australia	Rheumatic heart disease or acute rheumatic fever	2	Cross-sectional study	√	√
Shah *et al*, 2013^31^	UK	Diabetes, hypertension	1	Cohort study		√
Shelley *et al*, 2020^27^	US	No existing risk factor, smoking	2	Randomised controlled trial	√	√
Tu *et al*, 2017^18^	Canada	No existing risk factor, smoking	4	Literature review and modified Delphi process	√	√
van der Pol *et al*, 2019^32^	UK	Diabetes	1	Cohort study		√

^a^Risk of CVD was categorised based on Framingham risk equation or World Health Organization and International Society of Hypertension risk scores; high risk was identified based on the presence of at least three of the following risk factors: age (males aged ≥45 years, females aged ≥55 years), smoking, hypertension, and dyslipidaemia. Comma separates different conditions. ^b^A systematic review, countries not specified. ^c^Austria, Belgium, France, Germany, the Netherlands, Slovenia, Spain, Switzerland, and the UK. ^d^Austria, Belgium, Finland, France, Germany, the Netherlands, Slovenia, Spain, Switzerland, and the UK. CVD = cardiovascular disease. COPD = chronic obstructive pulmonary disease. NICE = National Institute for Health and Care Excellence. RACGP = Royal Australian College of General Practitioners.

The majority of these indicators were reported in high-income countries, mainly from Canada (26.7%; two articles)^18,19^ and Slovenia (20.0%; one article).^15^ A few indicators were reported from Tajikistan (2.2%; one article)^20^ and Switzerland (1.1%; one article).^21^ Around 16% of indicators were collected from two cross-sectional surveys in Europe.^22,35^ The quality indicators identified, with detailed descriptions of their numerators and denominators by domains and disease conditions or risk factors, are presented in Table 2. Overall, 47.8% (*n* = 43/90) of all the indicators identified did not have any specific recommendation on the reporting frequency as a part of their definition (see Supplementary Table S2).^15,18–20,24,26,28,29,33,35^

**Table 2.** Definition of strong methodological quality indicators for assessment or advice

Domains or conditions	Numerator	Denominator^a^	References
**Assessment**			
Body weight	Record of weight, height or waist circumference, or BMI	All patients aged ≥15 years All patients with diabetes All patients categorised as moderate or high risk of CVD	^15,18,19,22–25,35^
Smoking	Record of smoking status	All patients aged ≥15 years All past smokers All patients categorised as high risk of CVD All active patients aged 10–80 years All patients with any or any combination of CHD, PAD, stroke or TIA, hypertension, diabetes, COPD, CKD, asthma, schizophrenia, bipolar affective disorder or other psychoses All patients with hypertension or diabetes All patients on the hypertension register aged 16–74 years	^15,18–20,22–30,33,34^
Physical activity	Record of physical activity or status or intensity level	All patients categorised as high risk of CVD All patients on the hypertension register aged 16–74 years	^15,19,22,25,35^
Alcohol	Record of alcohol consumption status	All patients aged ≥15 years All patients categorised as moderate or high risk of CVD All patients aged 15–80 years	^19,23,29^
Healthy eating	Record of salty foods or salt consumption	All patients categorised as moderate or high risk of CVD	^19^
**Advice or education**			
Body weight	Record of ≥1 offered follow-up consultation on weight reduction	All patients categorised as moderate or high risk of CVD All patients with obesity or obese	^15,19,25^
Smoking	Recommendation for lifestyle changes to stop smoking	All patients aged >15 years All current smokers All patients categorised as moderate or high risk of CVD All patients with any or any combination of CHD, PAD, stroke or TIA, hypertension, diabetes, COPD, CKD, asthma, schizophrenia, bipolar affective disorder or other psychoses	^15,18,19,22,27,28,30,34,35^
Physical activity	Recommendation for lifestyle changes to increase physical activity	All patients categorised as moderate or high risk of CVD All patients on the hypertension register aged between 16 and 74 years All patients with diabetes	^15,19,22,25,35^
Alcohol	Recommendation for lifestyle changes to reduce alcohol consumption	All patients categorised as moderate or high risk of CVD	^15,19^
Healthy eating	Recommendation for lifestyle changes to reduce sodium intake	All patients categorised as moderate or high risk of CVD All patients with diabetes or hypertension All patients at risk of rheumatic heart disease or acute rheumatic fever	^15,19,22,35^
Immunisation	Record of influenza immunisation	All patients with diabetes All patients aged ≥65 years	^15,23,24,28,32^
General advice	Record of ≥1 offered specific advice on healthy lifestyle	All patients categorised as high risk of CVD	^15^

^a^Denominators are listed according to the recommendations for different contexts reported in the articles. Risk of CVD was categorised based on Framingham risk equation or World Health Organization and International Society of Hypertension risk scores; high risk was identified based on the presence of at least three of the following risk factors: age (males aged ≥45 years, females aged ≥55 years), smoking, hypertension, and dyslipidaemia. BMI = body mass index. CHD = coronary heart disease. CKD = chronic kidney disease. COPD = chronic obstructive pulmonary disease. CVD = cardiovascular disease. PAD = peripheral arterial disease. TIA = transient ischaemic attack.

### Summary of indicators on monitoring of lifestyle risk factors

Overall, one in five indicators reported were on assessment of smoking status (*n* = 19/90; 21.1%),^15,18,20,22–25,27–30,33–35^ followed by body weight (*n* = 16; 17.8%),^15,18,19,22–25,35^ advice on smoking cessation (*n* = 12; 13.3%),^15,18,19,22,27,28,30,34,35^ immunisation uptake (*n* = 8; 8.9%),^15,23,24,28,32^ healthy eating (*n* = 7; 7.8%),^15,19,22,35^ physical activity (*n* = 7; 7.8%),^15,19,22,25,35^ and assessment of physical activity (*n* = 6; 6.7%).^15,19,22,25,35^ Indicators on assessment of healthy eating (*n* = 2; 2.2%)^15,19,22,35^ and alcohol consumption (*n* = 3; 3.3%)^15,19^ were the least reported. Most of the indicators related to the assessment of lifestyle risk factors were on the assessment of smoking (*n* = 19/46; 41.3%),^15,18–20,22–29,33,34^ followed by body weight (*n* = 16; 34.8%).^15,18,19,22–25,35^ There were very few indicators related to assessment of alcohol consumption (*n* = 3; 6.5%)^19,23,29^ or healthy eating (*n* = 2; 4.3%).^19^ Indicators related to advice on healthy lifestyle were often smoking cessation (*n* = 12/44; 27.3%),^15,18,19,22,27,28,30,34,35^ advice on or uptake of immunisation (*n* = 8; 18.2%),^21,23,24,26,28,31,32^ physical activity (*n* = 7; 15.9%),^15,19,22,25,35^ or healthy eating (*n* = 7; 15.9%).^15,19,22,35^ Few articles included indicators for advice related to reduction of alcohol consumption (*n* = 3; 6.8%)^15,19^ or management of multiple risk factors (*n* = 1; 2.3%).^9^

**Figure 2.**
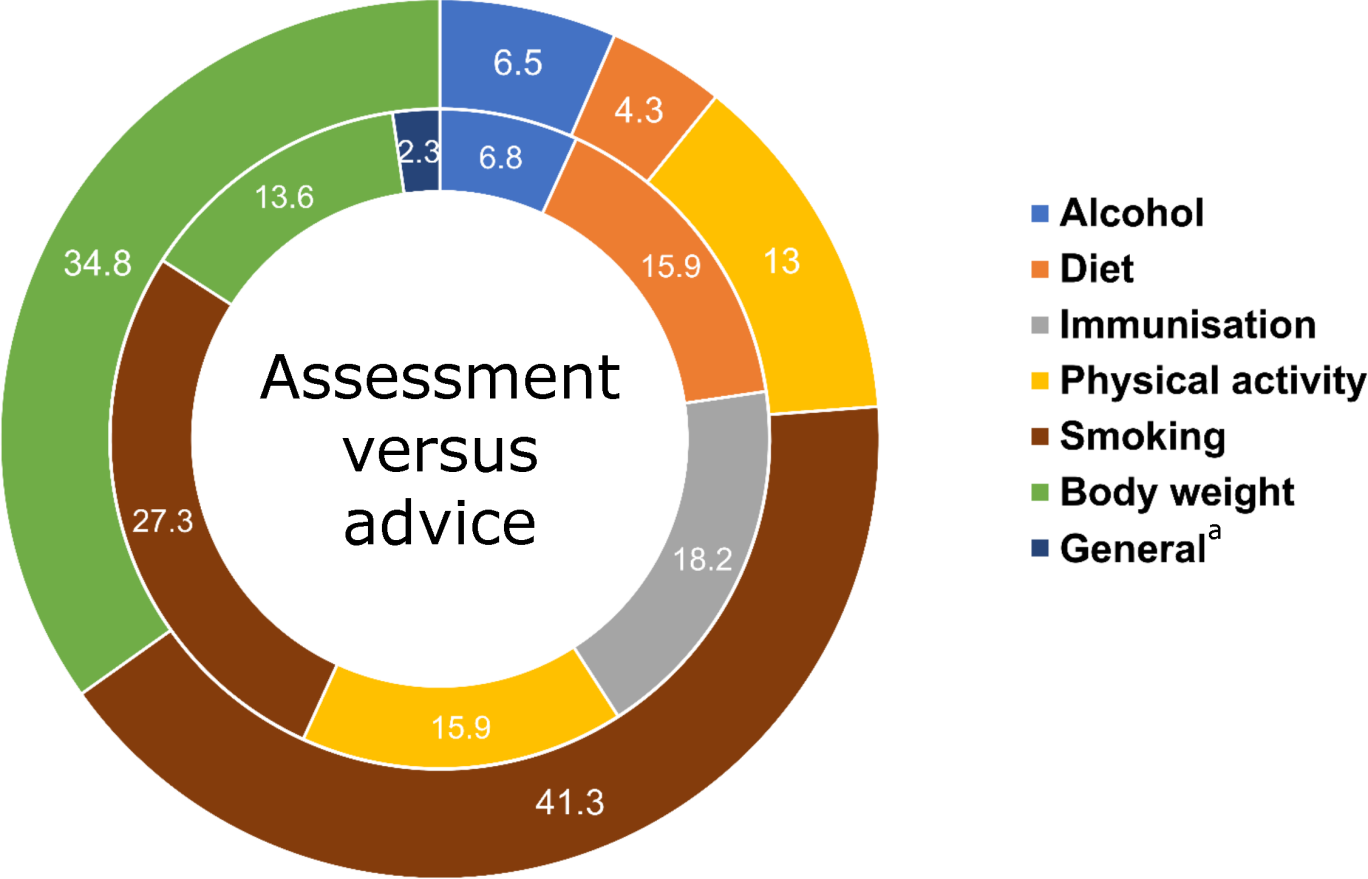
Proportions of indicators on assessment of lifestyle risk factors (outer circle; *n* = 46; 51.1%) versus advice on healthy lifestyle (inner circle; *n* = 44; 48.9%). ^a^Includes indicators related to generic lifestyle advice.

A comparison between the assessment of lifestyle risk factors versus advice on healthy lifestyle demonstrated gaps in monitoring lifestyle risk factors. There were more assessment-related indicators than advice-related ones for smoking (*n* = 19/46 [41.3%] assessment versus *n* = 12/44 [27.3%] advice) and body weight (*n* = 16/46 [34.8%] versus *n* = 6/44 [13.6%]). Interestingly, there were more indicators for advice on healthy eating (*n* = 7/44; 15.9%) than for assessing it (*n* = 2/46; 4.3%) (Figure 2).

### Quality indicators by the existing risk status or disease conditions

Half (*n* = 46/90; 51.1%) of the quality indicators identified were developed for populations at risk of CVD (categorised based on risk scoring or assessment of multiple risk factors),^15,19,22,25,28,35^ followed by those with existing disease condition(s) (*n* = 24; 26.7%),^15,18–26,28,31,32,35^ and those with no existing risk factor (*n* = 20; 22.2%).^15,18,21,23,26–31,35^ Indicators on lifestyle assessment or advice for people with specific conditions or risk factors were largely reported for diabetes (*n* = 9; 10.0%),^15,21,23,24,26,28,31,32^ followed by hypertension (*n* = 8; 8.9%)^15,20,24,25,31,34^ or obesity (*n* = 2; 2.2%).^15,19^ (Table 3). Indicators on the assessment of body weight were often monitored in people at risk of CVD (43.8%).^15,18,19,22–25,35^ Quality indicators for the assessment of body weight for those with hypertension or obesity were lacking.

**Table 3.** Quality indicators on lifestyle management by conditions (*n* = 19 articles with 90 indicators)

Conditions	Assessment of lifestyle risk factors, *n* (%)					Advice or education on healthy lifestyle, *n* (%)						
	Body weight (*n* = 16)	Smoking (*n* = 19)	Physical activity (*n* = 6)	Alcohol consumption (*n* = 3)	Healthy eating (*n* = 2)	Weight control (*n* = 6)	Smoking cessation (*n* = 12)	Physical activity (*n* = 7)	Low-level alcohol consumption (*n* = 3)	Healthy eating (*n* = 7)	Uptake of immunisation (*n* = 8)	General^a^ (*n* = 1)
No existing risk factor	7 (43.8)	6 (31.6)	2 (33.3)	2 (66.7)	—	—	2 (16.7)	—	—	—	1 (12.5)	-
At risk of CVD^b^	7 (43.8)	7 (36.8)	3 (50.0)	1 (33.3)	2 (100.0)	4 (66.7)	7 (58.3)	5 (71.4)	3 (100.0)	5 (71.4)	1 (12.5)	1 (100.0)
High risk of CVD	2 (12.5)	4 (21.1)	2 (33.3)	*—*	*—*	1 (16.7)	4 (33.3)	2 (28.6)	1 (33.3)	3 (42.9)	*—*	1 (100.0)
Moderate or high risk of CVD	5 (31.3)	3 (15.8)	1 (16.7)	1 (33.3)	2 (100.0)	3 (50.0)	2 (16.7)	3 (42.9)	2 (66.7)	2 (28.6)	*—*	*-*
Any risk category of CVD^c^	*—*	*—*	*—*	*—*	*—*	*—*	1 (8.3)	*—*	*—*	*—*	1 (12.5)	*-*
Hypertension^d^	—	4 (21.1)	1 (16.7)	—	—	—	—	1 (14.3)	—	1 (14.3)	1 (12.5)	-
Diabetes^d^	2 (12.5)	1 (5.3)	—	—	—	—	—	1 (14.3)	—	1 (14.3)	4 (50.0)	-
Obesity	—	—	—	—	—	2 (33.3)	—	—	—	—	—	-
Smokers	—	—	—	—	—	—	3 (25.0)	—	—	—	—	-
Other^e^	—	1 (5.3)	—	—	—	—	—	—	—	—	1 (12.5)	-

^a^General recommendations includes advice on the management of multiple risk factors. ^b^Risk of CVD was categorised based on Framingham risk equation or World Health Organization and International Society of Hypertension risk scores; high risk was identified based on the presence of at least three of the following risk factors: age (males aged ≥45 years, females aged ≥55 years), smoking, hypertension, and dyslipidaemia. ^c^Risk category not reported, indicators reported for people at risk of CVD or persons aged ≥65 years. ^d^Single indicator overlapped with assessment and advice for diabetes and hypertension. ^e^Other conditions include: rheumatic heart disease or acute rheumatic fever. — = indicator not reported. CVD = cardiovascular disease.

Fifty per cent of indicators on physical activity were focused on populations at risk of CVD,^15,19,22,25,35^ with no specific reference to people with diabetes or obesity. Indicators on alcohol consumption were lacking for those with existing conditions, such as hypertension, diabetes, and obesity. The majority of advice on healthy lifestyle management indicators was lacking for those with obesity, no existing risk factors, and smokers. Advice on alcohol consumption was reported only for those at risk of CVD. Notably, advice on or uptake of influenza immunisation was largely reported for patients with diabetes for the primary prevention of CVD (Table 3).

## Discussion

### Summary

In this review, we identified and summarised indicators being used for monitoring lifestyle risk factors around the world, developed through a strong methodological quality to enable easy adaptation within the country context. Smoking, body weight, and physical activity were the most frequently reported, with over half of the indicators addressing these lifestyle risk factors. Indicators related to healthy eating and alcohol consumption were the least frequently reported. There were gaps in assessment of lifestyle risk factors versus advice on healthy lifestyle for smoking, body weight, and healthy eating. Additionally, we found that nearly half of the indicators lacked specific frequency for the monitoring of lifestyle risk factors.

### Strengths and limitations

To the best of our knowledge, this is the first review focusing on monitoring of indicators on lifestyle management for the primary prevention of CVD in primary care. The indicators identified in this review will enable greater adoption and standardisation for monitoring of lifestyle risk factors in primary care. However, it is essential to acknowledge the limitations of this review. First, given that this review was limited to articles or literature with strong methodological quality, there is a possibility of missing relevant indicators reported in articles or literature with lower methodological quality. Second, focusing only on articles or literature published in the English language means that we may have missed some indicators from non-English speaking countries. This review may not be fully applicable to non-English-speaking contexts, emphasising the critical need for future review to be more inclusive and representative of diverse populations.

### Comparison with existing literature

Contemporary clinical practice guidelines strongly recommend adopting a healthy lifestyle as a first-line recommendation for the primary prevention of CVD.^36–40^ However, we found that monitoring and reporting of lifestyle risk factors using indicators is sub-optimal, predominantly focused on smoking status with limited emphasis on other important risk factors, such as physical inactivity, unhealthy diet, or alcohol consumption. These gaps could be owing to the lack of a clear mandate or guideline on the assessment or advice on healthy lifestyle, the lack of a field to record lifestyle risk factors in electronic health records, or the lack of incentives for assessing lifestyle risk factors in primary care and documenting their management.^35,41–46^

Studies have reported that intensive behavioural or lifestyle counselling improves health outcomes (for example, reduction in blood cholesterol or systolic blood pressure) among those with established cardiovascular risk factors.^47^ However, in our review, indicators of advice on healthy lifestyle were minimal for patients with existing risk factors such as hypertension or diabetes. Less reporting could be owing to the lack of indicators or advice provided as part of general counselling. Therefore, it is crucial to monitor and report advice on healthy lifestyle in primary care records to improve counselling on healthy lifestyle. Usually, cardiometabolic risk factors or medication prescriptions are well documented, and a similar approach is needed for documenting lifestyle risk factor advice.^43,45^

We found gaps when comparing the assessment of lifestyle risk factors versus advice on healthy lifestyle. There were more assessment indicators than advice-related indicators for smoking and body weight. Studies from Australia,^8,43^ the US,^44^ and the UK^42,45^ highlighted the importance of monitoring and prioritising advice on healthy lifestyle (for example, physical activity) for behaviour change. Therefore, lifestyle risk factors need to be monitored with an appropriate balance of assessment and advice-related indicators in primary care.

Data on lifestyle risk factors are collected mainly through population-based surveys or as part of lifestyle intervention studies for research or population-level monitoring purposes.^43–46^ Therefore, there is a need for real-time data on monitoring of lifestyle risk factors at the individual level to track their behaviours, follow-up with tailored messages, and ultimately sustain their behaviour change. Real-time data on monitoring and reporting lifestyle risk factors could be improved through a multidisciplinary approach, involving professionals such as nurses, dietitians, and psychologists to enhance lifestyle management monitoring, promoting a holistic and patient-centred approach.^48^

### Implications for research and practice

These strong methodological quality measurable indicators can be customised and adapted according to country-specific clinical practice guidelines, considering socioeconomic and cultural context, comorbidities, and at-risk populations.^49^ There is a need to develop indicators with an appropriate mix of assessment (for example, assessment of physical activity) and advice (for example, advice on physical activity) indicators among individuals with specific risk or disease conditions such as obesity, current smokers, chronic kidney disease, hypertension, and diabetes.

We found that nearly half of the indicators lacked information on the frequency of monitoring as a part of their indicator definition. It is essential to standardise these indicators with a defined frequency of monitoring (for example, every visit, weekly, or monthly) so the quality of lifestyle management in primary care can be tracked at a patient level. Including lifestyle indicators for both assessment and advice will enable routine monitoring of the effectiveness of lifestyle interventions over time.

In conclusion, we summarised quality indicators on assessment and advice for lifestyle management in primary care with strong methodological quality. These indicators can inform the standardisation and contextualisation for routine monitoring of lifestyle risk factors across countries. There is a need to have a balanced mix of assessment and advice-related indicators for comprehensive tracking of lifestyle risk factors. Future research should focus on validating, adapting, or tailoring these indicators to individual country’s context.^12^

## Data

All cited articles are publicly available.
